# Encapsulation of 16-Hydroxycleroda-3,13-Dine-16,15-Olide in Mesoporous Silica Nanoparticles as a Natural Dipeptidyl Peptidase-4 Inhibitor Potentiated Hypoglycemia in Diabetic Mice

**DOI:** 10.3390/nano7050112

**Published:** 2017-05-12

**Authors:** Po-Kai Huang, Shi-Xiang Lin, May-Jywan Tsai, Max K. Leong, Shian-Ren Lin, Ranjith Kumar Kankala, Chia-Hung Lee, Ching-Feng Weng

**Affiliations:** 1 Department of Life Science and Institute of Biotechnology, National Dong Hwa University, Hualien 97401, Taiwan; kevin7699402@hotmail.com (P.-K.H.); scujameslin@hotmail.com (S.-X.L.); d9813003@gms.ndhu.edu.tw (S.-R.L.); 2 Neural Regeneration Laboratory, Neurological Institute, Taipei Veterans General Hospital, Taipei 11217, Taiwan; mjtsai2@vghtpe.gov.tw; 3 Department of Chemistry, National Dong Hwa University, Hualien 97401, Taiwan; leong@gms.ndhu.edu.tw; 4 College of Chemical Engineering, Huaqiao University, Xiamen 361021, China; ranjithkankala@hqu.edu.cn

**Keywords:** diabetes, dipeptidyl peptidase-4, glucagon-like peptide-1 (GLP-1), insulin resistance, glycated hemoglobin (HbA1c), mesoporous silica nanoparticles

## Abstract

Natural supplements comprise good efficacy with less adverse effects as against diabetic therapy, but their advancement as anti-diabetic agents is unsatisfactory with regard to the delivery system. Dipeptidyl peptidase-4 (DPP4)/CD26) can degrade glucagon-like pepetide-1 (GLP-1) which renders a decrease of blood glucose levels. 16-hydroxycleroda-3,13-dine-16,15-olide (HCD) extracted from Polyalthia longifolia, exhibits numerous medicinal potentials including hypoglycemic potential. On consideration of HCD application, the bioavailability is affected by low solubility. Extended experiments of anti-diabetic efficacy confirmed HCD biocompatible with mesoporous silica nanoparticles (MSNs) encapsulation resulted in a sustained release property in delivering HCD for the inhibition of DPP4 via the activity and protein levels of DPP4 analysis. In the enzymatic activity assay, MSN-HCD directly changed DPP4 activity. Moreover, MSN-HCD nanoparticles were treated with Caco-2 cells and the protein levels of DPP4 determined within the cells. The results revealed that MSN-HCD caused reduction of DPP4 activity in a time- and dose-dependent fashion. Orally administered MSN-HCD in diet-induced diabetic mice alleviated blood glucose via an oral glucose tolerance test. In addition, administration of MSN-HCD for five weeks revealed that the biochemical cues such as pyruvate transaminase (GPT), glutamate oxaloacetate transaminase (GOT), triglycerides (TG), cholesterol (CHO), and glycated hemoglobin (HbA1c) in mice were commendable as further confirmation of MSN-HCD efficacy and less adverse effects in down-regulation of hyperglycemia. Furthermore, this formulation effectively controlled blood glucose and significantly decreased the body weight of mice, suggesting that MSN-HCD exerts natural DPP4 inhibitor as a potential clinical drug for the treatment of diabetes.

## 1. Introduction

Diabetes mellitus (DM) is a current major public health problem worldwide because approximately one in every five adults is diagnosed with it. It is the most prominent metabolic disorder with serious complexity that occurs due to increases in blood glucose levels caused by pancreatic failure to release insulin or resistance from body tissues. Conventional treatments include regular glucose monitoring or else directly administered insulin [1]. Currently, most treatments are dependent on insulin based delivery, which is typically self-regulated glucose sensitive insulin release from embedded matrix systems possessing few enzymes [1,2]. In addition to successful delivery of insulin, self-administration of insulin can result in hypoglycemia with consequences such as unconsciousness, coma, and brain damage, etc. The alternative approach to treat this disorder is by sensitizing pancreatic cells for effective treatment of hyperglycemic conditions through insulin release. Proglucagon-derived peptides including glucagon-like peptide-1 (GLP-1) and 2 (GLP-2) are secreted from an L cell. GLP-1 is a protein peptide with 37 amino acids and one of the main incretin hormones [3,4] possessing extra-pancreatic effects, such as delayed gastric emptying [5], appetite suppression [6], increase insulin sensitivity, and insulin secretion [7]. Unfortunately, the secretion of GLP-1 is impaired in type-2 diabetic mellitus (T2 DM). Nevertheless, it is normal in the first-degree relatives of diabetic patients. This indicates that the reduction of GLP-1 in T2 DM patients is a consequence rather than a cause of diabetes [8,9,10]. Recently, GLP-1 has been proved to comprise a therapeutic potential for T2 DM treatment. Earlier, Sitagliptin, an oral hypoglycemic agent that acts tby inhibiting dipeptidyl peptidase-4 (DPP4) enzyme, can raise two incretin hormone (GLP-1 and GIP) concentrations. They both increase the sensitivity of pancreatic cells to synthesize and release insulin. Additionally, its side effects include nasopharyngitis, upper respiratory tract infection, abdominal pain, and headache [11] which limits its applicability.

Amongst inorganic nanomaterials, mesoporous silica nanoparticles (MSNs) have attracted considerable attention due to their great utility in various fields specifically for drug delivery. These carriers encompass a unique mesoporous structure, i.e., large surface area and pore volume, with tunable pore size. Different exposure routes of MSN alone in vivo showed low toxicity through oral administration [12]. Also, they provide great potential in the loading of drug molecules. They have a high affinity to hold drug cargo in the pore channels and are able to release them as directed, which enables better control of release kinetics that can improve oral bioavailability as well as serve as an efficient drug delivery system [13]. These biocompatible nano-carriers are smart, feasible and convenient for chemical functionalization of a surface [14], for targeting ability and controlled delivery for various therapeutic purposes such as in cancer [15,16,17,18,19], ischemia [20], inflammation [21], bacterial infections [22], other factors such as imaging [23,24], and biomedical applications [25]. In addition to MSNs, numerous other nanocarriers and their hybrids have also attracted the attention of researchers for various biomedical applications [26,27,28,29,30,31,32]. A revolutionary progress for drug delivery and a targeting process at lab scale have been instigated. The new hope is that this can change the landscape of the pharmaceutical industry in the future [24].

Recently, a few researchers have made progress on anti-diabetic therapies using MSNs; however, their approaches are limited to insulin delivery to treat insulin based diabetes by controlled delivery or utilizing MSNs as sensors. One report revealed self-regulated insulin delivery systems with pH-gated, glucose-sensitive MSN and polymers as core-shell nano-hybrids for controlled delivery. The controlled release of insulin is dependent on a polymeric shell and extrinsic glucose concentrations [33]. The hybrid nanoparticles have good biocompatibility, high loading capacity, and control insulin release. Interestingly, one study demonstrated a smart system of alizarin complex one functionalized MSNs for glucose responsive delivery of multiple drugs and their real-time monitoring for accurate quantification [34]. However, MSN-conjugated natural compound as an anti-diabetic reagent remains unexplored. Based on literature reviews, the natural compound 16-Hydroxycleroda-3,13-dien-15,16-olide (HCD) isolated from Polyalthia longifolia bark has been unveiled as showing various strong anti-inflammatory activities [35,36,37] and further revealed the induction of CML K562 cell apoptosis via alteration of histone-modifying enzymes PRC2 complex expression [38]. Nonetheless, this promising drug candidate is limited in its applicability due to poor solubility in water, which results in poor clinical bioavailability. Reports have elucidated that approximately 70% of new chemical entities (NCIs) and around 40% of currently marketed oral drugs are poorly soluble in water [39,40]. Thereby, improving solubility of these NCIs becomes a crucial issue in the formulation process during new drug development [41].

Herewith, we have designed the surface functionalization of MSNs with amine groups to serve as reservoirs for effective immobilization of bulk natural compounds into pores for anti-diabetic therapies. The combination of the advantages of MSN properties controlled delivery potential with the anti-diabetic therapeutic ability of natural compounds was employed to develop a novel carrier that effectively treats diabetes. Firstly, the synthesized nano-carriers were physically characterized and further, anti-diabetic therapy both in vitro through Western blot for protein expression, DPP4 inhibition assay and in vivo through glucose tolerance as well as glucose intolerance tests in diet-induced diabetic mice were investigated. We strongly anticipate that these delivery systems using MSNs can be promising carriers for natural compounds to treat diabetes.

## 2. Results

### 2.1. Physical Characterizations

**Figure 1:**
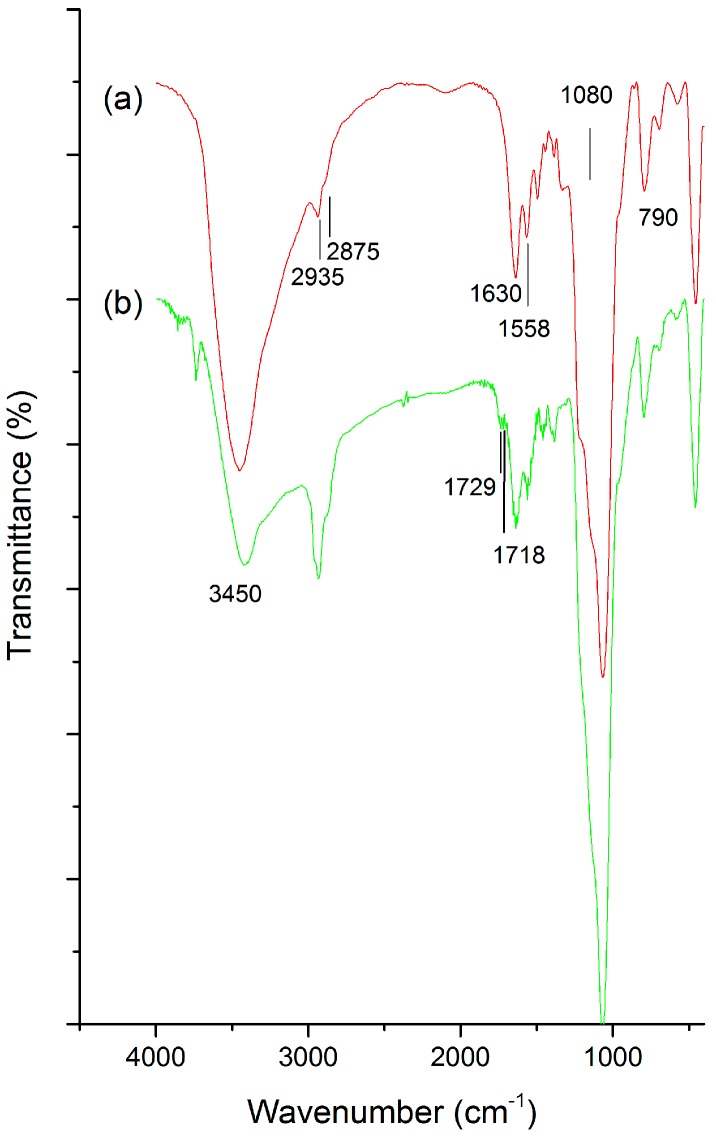
Fourier transform infra-red (FT-IR) spectra of (**a**) mesoporous silica nanoparticles (MSN)-NH_2_; and (**b**) MSN-HCD. (16-hydroxycleroda-3,13-dine-16,15-olide, HCD).

Figure 1 illustrates the characterization of chemical bonds and attached organic transformations using Fourier transform infra-red (FT-IR) spectra of various MSN conjugates. The peaks at 1080, 790 cm^–1^ represent Si–O–Si groups, along with the strong band (3100–3700 cm^–1^) and a peak at 1630 cm^–1^ from O–H stretch and deformation respectively, of the absorbed molecular H_2_O and surface hydroxyl groups of the silica framework (Figure 1a). The C–H stretch peaks at 2935 and 2875 cm^–1^ evidence the post-modification of amine groups in the pores (Figure 1a, b). In addition, a sharp peak at 1558 cm^–1^ represents N–H stretch confirming successful surface functionalization (Figure 1a). In comparison to the above functionalization, a new peak at 1718 cm^–1^ reflecting the carbonyl group of HCD is attached to MSN-NH_2_ (Figure 1b).

**Figure 2:**
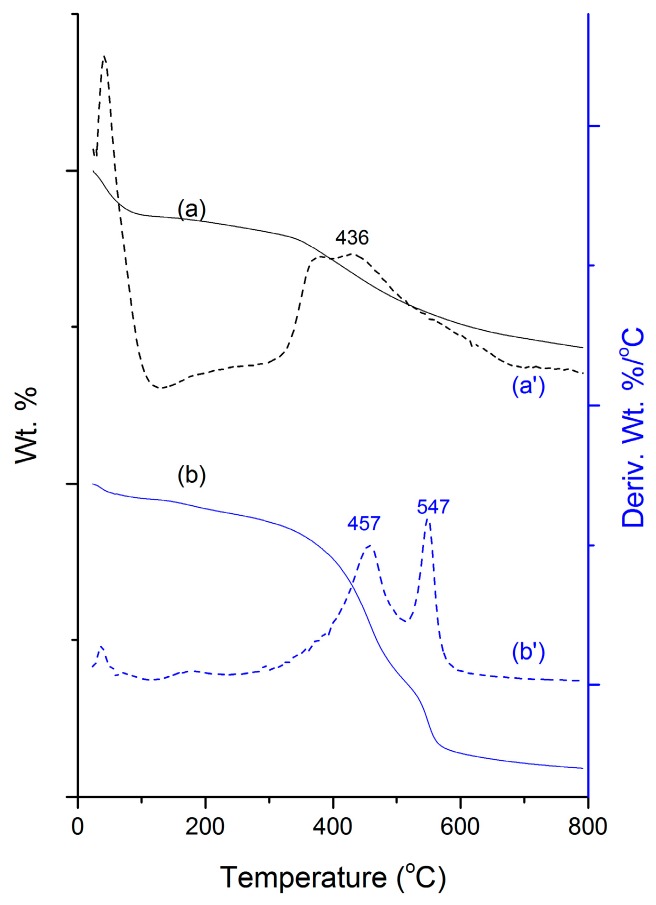
Thermogravimetric analysis (TGA) spectra of (**a**) MSN-NH_2_; and (**b**) MSN-HCD and their respective derivative weight loss curves (**a′**,**b′**). Mesoporous silica nanoparticles (MSN)-NH_2_; 16-hydroxycleroda-3,13-dine-16,15-olide (HCD).

The thermal properties of various conjugates were corroborated by thermogravimetric (TGA) analysis showing weight loss (Figure 2a,b) as well as derivative weight loss curves (Figure 2a′,b′). These data represent loading efficiency and thermal stability. The first event of weight loss in all the samples represents absorbed water on the surface of the sample at a temperature lower than 100 °C. The post-modified sample evidenced an additional broader weight loss event at a temperature range of 300–650 °C ascribed to successful organo-amine alteration in the silica framework (Figure 2a,a′). Furthermore, the weight loss of HCD-immobilized MSNs resulted in extra peaks displaying the fact that the HCD was effectively incorporated. In addition, the degradation temperature of HCD (Figure 2b,b′) in MSNs shifted towards the right.

**Figure 3:**
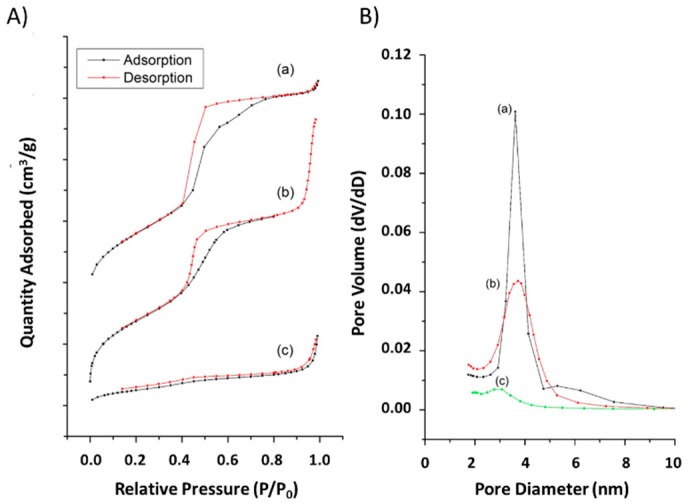
Nitrogen adsorption–desorption isotherms (**A**) and their respective distribution curves of Barret-Joyner-Halenda (BJH) pore size (**B**) of MSNs and conjugated-MSNs. (**a**) MSN; (**b**) MSN-NH_2_; and (**c**) MSN-HCD. Mesoporous silica nanoparticles (MSN)-NH_2_; 16-hydroxycleroda-3,13-dine-16,15-olide (HCD).

The dynamic light scatter (DLS) measurements illustrate hydrodynamic diameters in particle size distribution and the ζ-potential values (measured after dispersion in dd-H_2_O at pH 7.4) of amine-modified MSNs and their successive drug incorporated sample (Table 1). The average hydrodynamic diameter of amine group modified samples and subsequent HCD loading resulted in 168 ± 4.5 and 258 ± 5.7 nm, respectively. Actually, the average size of MSNs measured by DLS is slightly larger due to aggregation during measurement. The positive charge mesoporous silica matrix possesses strong electrostatic interactions with a negatively charged drug; i.e., HCD results in a negative shift in the zeta value and lands at +4 ± 0.3 mV (Table 1). Significant changes in structural properties (final Brunauer-Emmett-Teller (BET) surface area, pore volume, and pore size) within each modification measured by N_2_ adsorption–desorption isotherm curves were found (Figure 3, Table 1). The recorded BET isotherms exhibited type-IV per IUPAC classification. Post-modification of MSNs resulted in a lower final BET surface area of 855 m^2^/g than the MSNs (data not shown) representing the occupancy of amine-containing organosilane moieties in the pores. Further, with the immobilization of HCD, the final surface area drastically dropped down to 194 m^2^/g, suggesting a high amount of immobilized-HCD for amine-modification inside the pores (Figure 3A). In addition, the pore volume of the respective samples was reduced to 1.3 cm^3^/g and concomitantly to 0.4 cm^3^/g after amine-modification and HCD-immobilization, respectively.

### 2.2. In Vitro Assay of MSN-HCD

#### 2.2.1. The Inhibition of DPP4 Enzyme Activity

**Figure 4:**
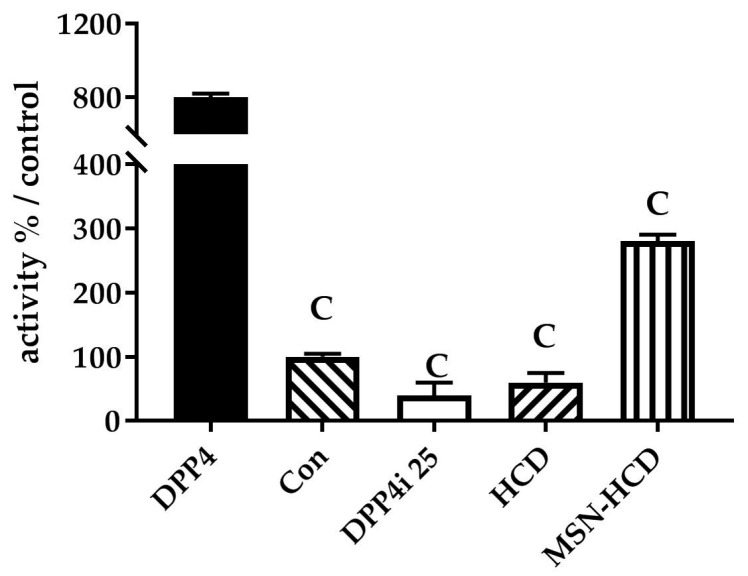
The inhibition of DPP4 enzyme activity. Con (P32/98, DPP4 inhibitor), DPP4i (Sitagliptin, clinical DPP4 inhibitor), HCD, and MSN-HCD. Data are expressed as means with standard deviations (mean ± SD). C, *p* < 0.001 treatment vs. DPP4 with one-way ANOVA. Mesoporous silica nanoparticles (MSN)-NH_2_; 16-hydroxycleroda-3,13-dine-16,15-olide (HCD).

Primarily, the inhibition of DPP4 enzyme activity was confirmed by various treatments according to parallel treatments simultaneously antagonized using clinical DPP4 inhibitor, Sitagliptin, and Con (P32/98, DPP4 inhibitor). We observed that pure HCD and nano-constructs conjugated HCD results in the inhibition of DPP4 enzyme according to the control (Figure 4).

#### 2.2.2. In Vitro Enzyme Activity of DPP4

**Figure 5:**
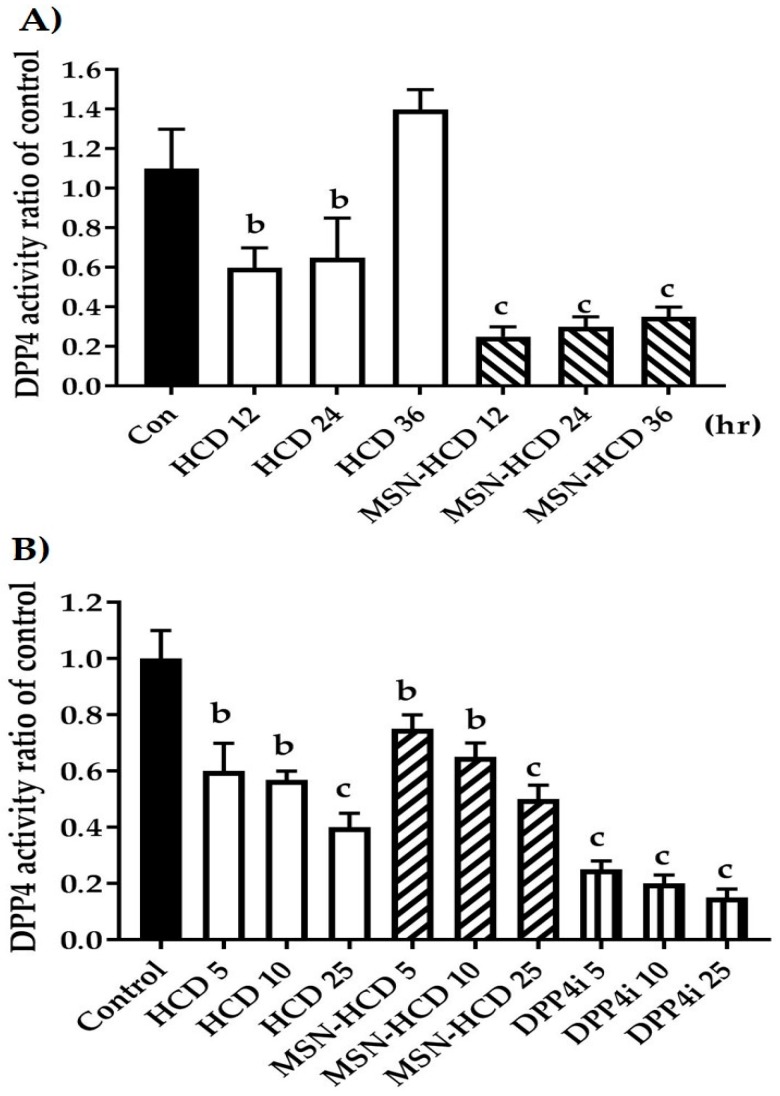
DPP4 activity in Caco-2 cells after HCD and MSN-HCD treatments. (**A**) Time-dependent inhibition of DPP4 protein in Caco-2 cells (in vitro). The designed formulation MSN-HCD (18.75 µg/mL) along with pure HCD (18.75 µg/mL) were compared with the control for 12, 24, and 36 h treatments; (**B**) Dose-dependent inhibition of DPP4 protein in Caco-2 cells (in vitro). The various concentrations of designed formulation MSN-HCD (5, 10, or 25 µg/mL) along with pure HCD (5, 10, or 25 µg/mL) were compared with the control and a clinical DPP4 inhibitor (5, 10, or 25 µg/mL, DPP4i-Sitagliptin). Data are expressed as mean ± SD. b, c, *p* < 0.01 or *p* < 0.001 treatment vs. control with one-way ANOVA. Mesoporous silica nanoparticles (MSN)-NH_2_; 16-hydroxycleroda-3,13-dine-16,15-olide (HCD).

Previously, the non-cytotoxicity of HCD concentration was determined as high as 30 µg/mL of HCD in Caco-2 cells (data not shown). Based on this concentration, the in vitro DPP4 inhibition of the designed formulation was extended robustly in a time-dependent fashion (Figure 5A) as well as in a dose-dependent manner (Figure 5B) in Caco-2 cells. Time-dependent inhibitions of DPP4 by MSN-HCD were effective a factor of 5-fold of pure HCD which was approximately 2-fold of the control. The inhibitory effect was reversed with increasing time from 12–36 h; however, efficacy of MSN-HCD was better when compared to the control and HCD alone (Figure 5A). For HCD treatment 36 h, DPP4 activity increased, speculating that this might be due to cell proliferation. In addition, the increase in the dose of the formulation resulted in highly effective inhibition of DPP4 in a dose-dependent manner (Figure 5B), but not much more effective than that of the clinic inhibitor (DPP4i-Sitagliptin).

### 2.3. In Vivo Test of MSN-HCD

#### 2.3.1. Hypoglycemic Effect via Long Term Administrations

**Figure 6:**
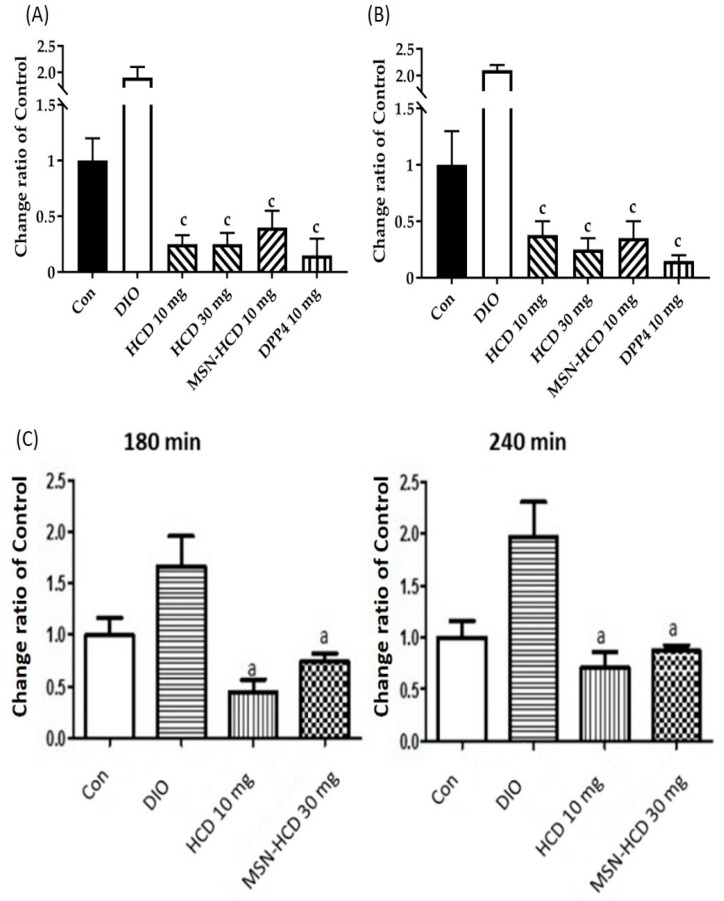
The alterations of blood glucose levels in mice after various treatments. High-fat diet and 60% fructose induced diabetic mice were treated with Con (control, *n* = 5), DIO (diet-induced obese, *n* = 6), 10 or 30 mg/kg B. wt. HCD (*n* = 6), 10 mg/kg B. wt. MSN-HCD (MSN conjugated HCD; 10 mg/kg B. wt., *n* = 6), and DPP4i (Sitagliptin, clinical DPP IV inhibitor; 10 mg/kg B. wt., *n* = 6) for 5 weeks (**A**) after fasting 12 h to measure the level of blood glucose. The blood glucose of Con (normal) and DIO groups are 93 ± 10 mg/dL and 225 ± 15 mg/dL, respectively. Also oral glucose tolerance test (**B**) for 180 min was performed to measure the change of blood glucose. The area under the curve (AUC) is calculated by area of time points (30 min each) from 0 to 180 min. (**C**) Oral glucose tolerance test measured at total 180 and 240 min after subjected to various treatments (Con, control, *n* = 5) and DIO (*n* = 6), HCD (10 mg/kg B. wt., *n* = 6), and MSN-HCD (30 mg/kg B. wt., *n* = 6). The AUC is calculated by area of time points (30 min each) from 0 to 180 or 240 min. Data are expressed as mean ± SD. a, *p* < 0.05; c, *p* < 0.001 treatment vs. DIO with one-way ANOVA. Mesoporous silica nanoparticles (MSN)-NH_2_; 16-hydroxycleroda-3,13-dine-16,15-olide (HCD).

High-fat diet with 60% fructose water induced diabetic mice were treated with Con (control, *n* = 5); DIO (diet-induced obese, *n* = 6); 10 or 30 mg/kg B. wt. of HCD (*n* = 6); 10 mg/kg B. wt. of MSN-HCD (MSN conjugated HCD, *n* = 6); and 10 mg/kg B. wt. of DPP4i (Sitagliptin, clinical DPP IV inhibitor, *n* = 6) for 5 weeks. Figure 6A depicts the natural compounds (MSN-HCD and HCD), the treated group resulted in lower fasting glucose levels as compared to DIO and CON after 12 h fasting.

#### 2.3.2. In Vivo Oral Glucose Tolerance Test (OGTT)

Subsequently, the Oral Glucose Tolerance Test (OGTT) was employed in diabetic mice as a model to examine the hypoglycemic effect of tested compounds by the determination of AUC. Mice were orally given pure HCD (10 or 30 mg/kg B. wt.) or MSN-HCD (10 mg/kg B. wt.) in different groups along with glucose (2 g/kg B. wt.) to measure blood glucose values at various time points of 30 min intervals for 180 min. This result indicated that both natural compound alone and nanoparticles conjugated natural compound had hypoglycemic effects (Figure 6B) in diabetic mice induced by a high-fat diet with high fructose water. In addition, two points (1) only once given to mouse and (2) loading capacity of HCD are necessary to be considered, therefore high dosage of MSN-HCD was applied to the next test. When diabetic mice were orally given MSN-HCD (30 mg/kg B. wt.), or pure HCD (10 mg/kg B. wt.) in different groups along with glucose (2 g/kg B. wt.) to measure blood glucose values at various time points (from 0 min to 180 and 240 min). Figure 6C illustrates the natural compounds treated group resulted in higher AUC levels at total 240 min as compared to total 180 min, whereas the AUC levels at total 240 min in the MSN-HCD treated group was not higher compared to that of total after 180 min. The data showed that the AUC levels of the nanoparticles treated group were similar to the control (no treatment) group, but significant reductions in glucose levels when compared to DIO mice (induced group) at different time points, suggesting a sustained effect of MSN-HCD on lowering blood glucose of diabetic mice.

#### 2.3.3. Serum Biochemical Analysis

**Figure 7:**
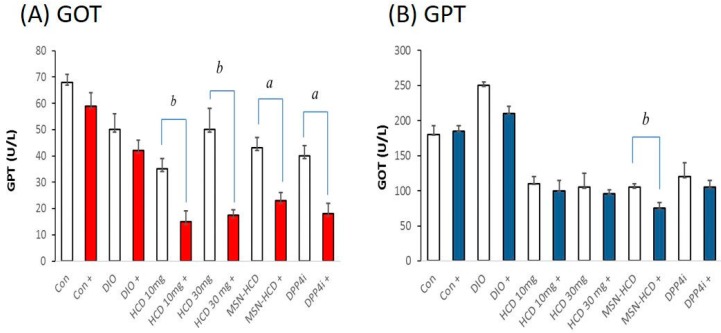

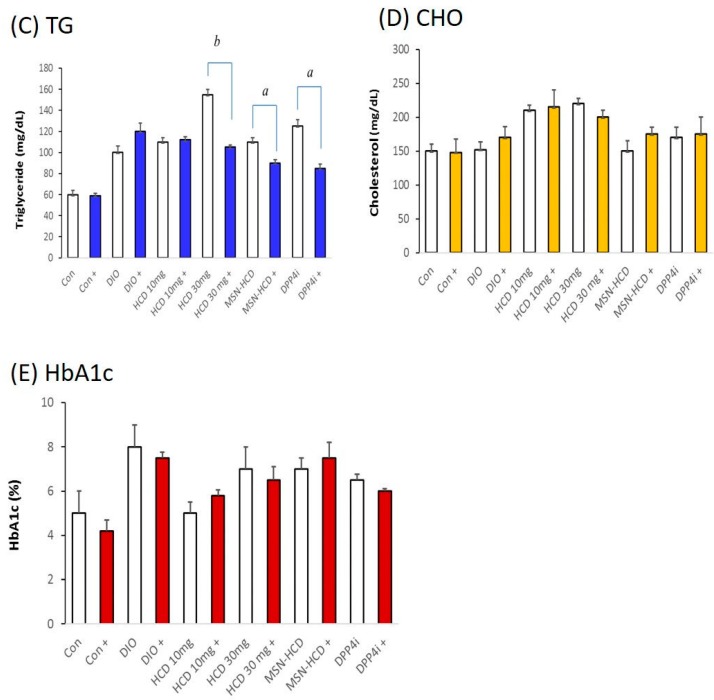
The changed levels of biochemical parameters. (**A**) glutamate oxaloacetate transaminase (GOT); (**B**) glutamate pyruvate transaminase (GPT); (**C**) triglyceride (TG); (**D**) cholesterol (CHO); and (**E**) glycated hemoglobin (HbA1c) in diabetes-induced mice for 5 weeks. When subjected to various treatments, Con (control, *n* = 5), DIO (diet-induced obese, *n* = 6), HCD (10 or 30 mg/kg B. wt., *n* = 6), MSN-HCD (10 mg/kg B. wt., *n* = 6) and DPP4i (10 mg/kg B. wt., *n* = 6). +: animal group after treatment. Data are expressed as mean ± SD. a, *p* < 0.05; b, *p* < 0.01 after treatment (+) vs. before treatment compared by pair *T*-test for each group. Mesoporous silica nanoparticles (MSN)-NH_2_; 16-hydroxycleroda-3,13-dine-16,15-olide (HCD).

After five weeks of treatments, blood was drawn from the subjects (mice) by piercing the cheek then serum biochemical values (GOT, GPT, TG, CHOL, and HbA1c) were measured. In order to monitor the individual difference in mice among various treated groups, a comparison was made between before and after administration of the designed formulation. The GOT levels were significantly lower than the other groups in MSN-HCDs (HCD conjugated MSNs) treatment, demonstrating HCD contained liver protective nature (Figure 7A). In addition, GPT levels were significantly reduced in all the treatment groups suggesting that long-term treatment had no significant adverse effect on liver function (Figure 7B). Low dosages of HCD alone and MSN-HCD are preferred to circumvent liver toxicity due to long-term treatment. In addition, sustained release drugs from the nanoparticles can also be considered. The TG and CHO levels of mice had no change in the normal group after the treatment as compared to the prior to treatment. Interestingly, while TG levels of mice were reduced after 30 mg/kg B.wt. HCD, 10 mg/kg B. wt. MSN-HCD, and 10 mg/kg B. wt. DPP4i treated for 5 weeks when compared to the accordingly prior to treatment (Figure 7C, D). Glycated hemoglobin (HbA1c) levels measured before and after administering HCD and its conjugated nanoparticle product resulted in no significant changes in both the cases (Figure 7E), demonstrating that the nano-carriers have almost no influence on the in vivo biochemical parameters.

#### 2.3.4. Body Weight Analysis

**Figure 8:**
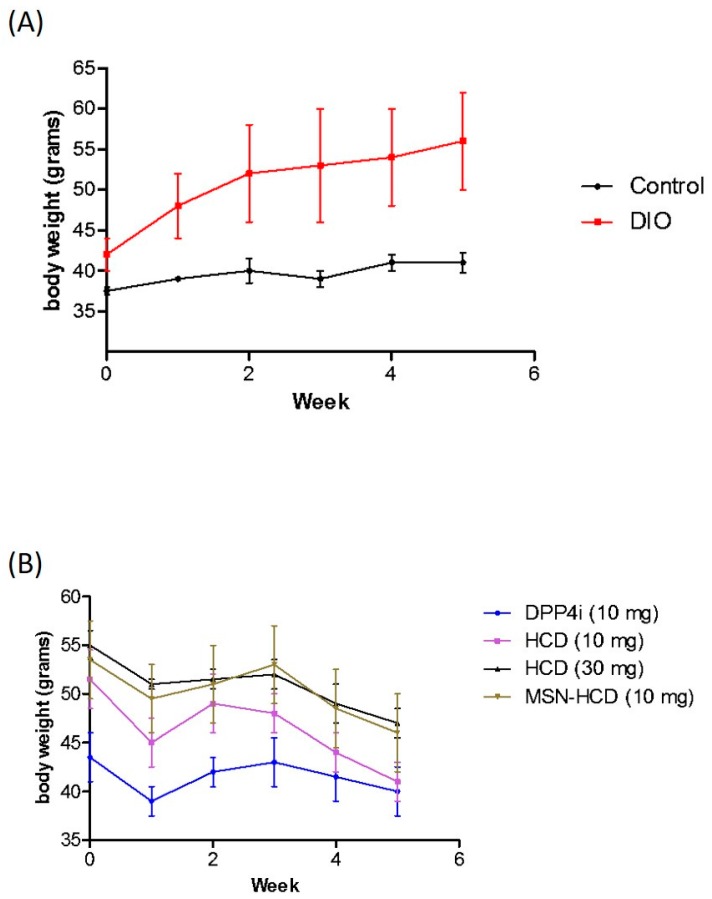
The body weight change of diabetic mice. (**A**) Control (*n* = 5) and DIO (diet-induced obese, *n* = 6); (**B**) Pure HCD (10 or 30 mg/kg B. wt., *n* = 6), MSN-HCD (10 mg/kg B. wt., *n* = 6), and DPP4i (10 mg/kg B. wt., *n* = 6) were administered to diabetes-induced mice for 5 weeks. Data are expressed as mean ± SD.

Diabetic mice were subjected to different treatment groups along with the normal mice as a control for five weeks (Figure 8). Surprisingly, the body weight of the various treatments in the beginning of the experimental period was different as seen in Figure 8B. It is noteworthy that insulin resistance could reduce the sensitivity of insulin and increase triglyceride hoarding, subsequently according to body weight. In the treatment groups, weight loss was an average of 4–6 g (Figure 8B) particular in HCD (10 or 30 mg/kg B. wt.) and MSN-HCD (10 mg/kg B. wt.), whereas the body weight of the non-treatment (Control and DIO) groups continued to increase (Figure 8A). These results agreed with the OGTT, where the treatment group had significantly lower AUC levels than the non-treatment group at total 180 min (Figure 7). After treatment, blood glucose and body metabolism were ameliorated and consequently the insulin resistance levels [4] decreased further the lowering of free fatty acids and triglyceride hoarding, which can achieve a decrease in body weight.

## 3. Discussion

The current investigations are discussed here in brief. Initially, we synthesized well-ordered hexagonal MSNs for incorporating a high payload of drug cargo. Subsequently, post-modification with silane resulted in an amine terminal, which is highly suitable to immobilize HCD. Furthermore, these nano-carriers were loaded with HCD and tested to resolve the complications associated with diabetes through a natural dipeptidase inhibitory way.

### 3.1. Physical Characterization

The FT-IR spectra of MSN (Figure 1a) reflect the surface silanol groups specifically, suggesting stable silica framework formation. Moreover, the peak intensity of aliphatic stretch higher in HCD immobilized MSNs when compared to that of MSN-NH_2_, confirmed its successful immobilization [41] through strong attractions with the amine-modified MSN surfaces. The surface charge of surfactant-extracted MSNs ensued with a negative charge (−35 mV) due to the silanol groups in the framework [41], which upon post-modification the MSNs resulted in having a positive charge, demonstrating enormous amine groups attached to the framework. This improved the affinity between drug and carrier, and subsequently increased drug loading efficiency. Post-grafting of amines plays a crucial role, where non-functionalized carriers result in physical loading through weak interactions and improper releasing profile [16,42]. Furthermore, the degradation temperature of HCD (Figure 2b,b′) in MSNs shifted towards the right (pure HCD degraded at around 285 °C) showing that the immobilization ensued inside the pores rather than on the surface, by which the stability of the sensitive drug improved after immobilization. We observed significant changes in structural properties (final BET surface area, pore volume, and pore size) within each modification in the MSN nanoconjugates [41] measured using N_2_ adsorption–desorption isotherm curves (Figure 3, Table 1). In a similar fashion, the pore volume of the respective samples was reduced after amine modification and HCD immobilization, respectively. From the characterization of these properties, it was confirmed that HCD was successfully encapsulated and further exerted hypoglycemic efficacy.

### 3.2. Hypoglycemic Effect of HCD Conjugated MSN

In the DPP4 enzymatic assay in vitro, pure HCD as well as HCD-MSN resulted in inhibition of DPP4 enzyme significantly in comparison with the control (Figure 4). The inhibition was both time-dependent as well as in a dose-dependent manner (Figure 5) in Caco-2 cells. Further confirmation was made by extending the study in in vivo; T2 DM mice were fed by a high-fat diet with 60% fructose in water to induce diabetes and the experimental results designated that the diabetic mice had insulin resistance, which instigated excess of free fatty acids in circulation. Of note, diet-induced diabetes is preferred fast and accurate to chemically-induced diabetes that the chances of animal death are high and it circumvents the risk of β-cell destruction. In the present study, diabetic mice were subjected to different treatment groups along with the normal mice as a control for five weeks. From OGTT assay of various periods (180 or 240 min), we found that MSN-HCD exerted a reducing effect on blood glucose and this lowering tendency was found at 240 min time point (Figure 6D). This result reveals that the sustained effect of MSN-HCD on lowering blood glucose could be attributed to slow release HCD from MSNs. After five weeks of treatment, blood was drawn from the subjects by piercing the cheek then serum biochemical values (GOT, GPT, TG, CHOL, and HbA1c) were recorded. These recordings suggest that the nano-carriers have almost no influence on the in vivo biochemical parameters (Figure 7), when comparing between prior to and post administration of the designed formulation. Surprisingly, the body weight of various treatments in the beginning of the experimental period was different as shown in Figure 8B. It is noteworthy that insulin resistance could reduce the sensitivity of insulin and increase triglyceride hoarding, subsequently responding to body weight. These results are in agreement with the OGTT, where the treatment group had significantly lower AUC levels than the non-treatment group at total 180 min (Figure 7). After treatment, blood glucose and body metabolism were ameliorated and consequently insulin resistance levels decreased further the lowering of free fatty acids and triglyceride hoarding, which can achieve a decrease in body weight. Based on these results, MSN-HCD provides potential for HCD delivery and slow release for controlling diabetes.

## 4. Materials and Methods

### 4.1. Materials

Natural products were isolated following the procedure as mentioned in the below section. All the chemicals, reagents, and solvents were purchased at a high purity grade. Cetyltrimethylammonium bromide (CTAB), ethylenediaminetetraacetic acid (EDTA), tetraethyl orthosilicate (TEOS) (98%), potassium phosphate monobasic (KH_2_PO_4_), potassium phosphate dibasic (K_2_HPO_4_), citric acid, ammonium hydroxide from Sigma-Aldrich Ltd. (St. Louis, MO, USA). The (3-aminopropyl) trimethoxysilane (APTS) and potassium bromide (KBr) (FT-IR grade) were obtained from Gelest (Morrisville, PA, USA), and Fisher Scientific Ltd. (Loughborough, UK), respectively.

### 4.2. Preparation of HCD Immobilized MSNs

#### 4.2.1. Preparation of MSNs

Hexagonal MSNs were synthesized at a low concentration of TEOS by a two-step preparation following the reported method [17]. Initially, CTAB (0.58 g) was added to ammonium hydroxide solution (0.51 M) and stirred for 30 min at 40 °C, then tetraethoxysilane (5 mL, 0.21 M) as well as structural swelling agent (n-octane) were added and stirred gently for 5 h. Further, TEOS (5 mL, 0.88 M) was added and stirred for an additional 2.5 h. Next, the solution mixture was aged at room temperature (RT) for 16 h and the MSNs were collected eventually by centrifuging at 12,000 rpm for 17 min. Surfactant was removed through a chemical extraction method to generate well-ordered pores to immobilize the drug cargo. The surfactant was removed by a chemical extraction method by refluxing the as-synthesized MSNs in acidic ethanol (30 mL of 99.5% ethanol and 1 g of 37% HCl) at 65 °C overnight. The extracted particles were washed three times with ethanol and stored in ethanol and used for post-modification.

#### 4.2.2. Post-Modification of MSNs (MSN-NH_2_)

Amine-modified MSNs was prepared by dispersing surfactant extracted nanoparticles (200 mg) in toluene (30 mL) for 30 min and 1 mL of APTS was added to the mixture. The contents were refluxed at 80 °C for 24 h. Later, the contents were centrifuged and washed three times with acetone and ethanol to remove the unconjugated saline and eventually suspended in ethanol for further drug loading.

#### 4.2.3. Isolation of Natural Compounds

The natural compound 16-hydroxycleroda-3,13-dine-16,15-olide (HCD) was isolated through an extraction process from the bark of Polyalthia longifolia var. The pendula Linn (Annonaceae), was kindly provided by Dr. Yi-Chen Chia (Department of Food Technology, Tajen University, Pingtung, Taiwan). The fresh leaves were placed in water (1 kg/1 L) and the mixture boiled until only 100 mL volume remained. The crude water extract was centrifuged at 3000 *g* for 12 min, and the supernatant was collected. Then, the supernatant was concentrated under reduced pressure and freeze-dried to yield powder (yield of 5%). The extracts were dissolved in a methanol–water (50:50 *v*/*v*) mobile phase mixture and separated using high performance liquid chromatography (HPLC) analysis. The identity of the compounds was fully determined by comparing their spectral data (Infra-Red (IR), Nuclear magnetic resonance (NMR), and Mass) spectrometry with information reported in the literature [36].

#### 4.2.4. Preparation of HCD Loaded MSN Samples (MSN-HCD)

Methanol was used as a solvent to load HCD effectively into MSNs to improve its solubility and loading efficiency [16]. Initially, the stock solution of HCD (100 mg in 5 mL of methanol) was prepared and added to the MSN-NH_2_ (100 mg) suspended in methanol. The resulted solution was vigorously stirred at RT for 24 h. Then, drug loaded nano-containers were centrifuged at 12,000 rpm for 20 min and washed twice with ethanol to remove the unconjugated-HCD.

### 4.3. In Vitro Test

#### 4.3.1. Cell Culture

Human colorectal adenocarcinoma (Caco-2) cell line was obtained from the Food Industry Research and Development Institute (FIRDI, Hsinchu, Taiwan). The Caco-2 cells were grown and maintained in Dulbecco’s Modified Eagle’s Medium (DMEM, GIBCO, Carlsbad, CA, USA) with high glucose containing 10% fetal bovine serum (FBS, GIBCO), DMEM containing 20% FBS with 2 mM of l-glutamine and DMEM (GIBCO) containing 20% FBS alone, respectively. An amount of 1% Penicillin/Streptomycin (GIBCO) was added to the media composition and maintained in a cell culture incubator (5% CO_2_ at 37 °C). Prior to the experiment, cells were seeded and cultured for 16–24 h.

#### 4.3.2. DPP4 Enzyme Activity

The enzymatic activity of DPP4 was performed using the assay kit (Enzo^®^ DPP4/CD26 Assay Kit for Biological Samples (BML-AK498)) possessing a chromogenic substrate (H-Gly-Pro-pNA) and a fluorogenic substrate (H-Gly-Pro-AMC). *p*-Nitroaniline (pNA) cleavage from the chromogenic substrate was correlated to the increased absorbance at 405 nm. This fluorometric assay is based on the cleavage of 7-amino-4-methylcoumarin (AMC) moiety from the C-terminus of the peptide substrate, which is correlated to its increased fluorescence intensity at 460 nm. First, 50 μL of assay buffer (50 mM Glycine, pH 8.7, 1 mM EDTA) was added into a 96-well clear microplate, next 20 μL of DPP4 enzyme (13 μU/μL) was added with 20 μL of candidate inhibitors, and finally 10 μL of pNA substrate (H-Gly-Pro-pNA, 5 mM) was added and the absorbance read at 405 nm by the ELISA plate reader (Thermo Labsystems, Opsys MR, Thermo-fisher scientific, Waltham, MA, USA).

#### 4.3.3. In Vitro DPP4 Inhibition Assay

The inhibition amount of DPP4 was detected by correlating its protein expression in Caco-2 cells. Lysis buffer (10 mM Tris-HCl, 150 mM NaCl, 0.04 U/mL aprotinin, 0.5% Nonidet P40, pH 8.0) was added to the cells for protein extraction and incubated at 4 °C for 1 h. Cells were centrifuged at 13,000 *g* at 4 °C for 30 min, the supernatant was quantified by Bradford protein assay (Bio-Rad, Hercules, CA, USA). An amount of 30 μg of sample protein was taken and then 70 μL of assay buffer was added into a 96-well clear microplate, next 10 μL of pNA substrate (H-Gly-Pro-pNA, 5 mM) was added and the absorbance read at 405 nm as abovementioned.

### 4.4. In Vivo Test

#### 4.4.1. Animals

Imprinting Control Region (ICR) mice were obtained National Laboratory Animal Center (Taipei, Taiwan) and maintained by providing controlled environmental conditions at room temperature (22 ± 2 °C) and humidity (60 ± 10%). The 12 h light and 12 h dark cycle were maintained throughout the study. Mice were well-maintained on a standard laboratory diet (carbohydrates; 60%, proteins; 28%, lipids; 12%, vitamins; 3%). In vivo experiments were performed following the regulations, “Guide for the Care and Use of Laboratory Animals” of National Dong-Hwa University with prior approval by the animal ethics committee, NDHU.

#### 4.4.2. Glucose Intolerance Induced

ICR male mice (6 weeks old) were fed a high-fat diet and 60% fructose water for 10 weeks. The high-fat diet comprised conventional chow (1 kg) plus lard (150 g) (23% of total saturated fatty acids and 77% of total unsaturated fatty acids, Chinshan oil, Wei Lih Foods Co., Changhua, Taiwan).

#### 4.4.3. Diabetes Test

Diet-induced ICR male mice were oral gavaged (p.o.) with d-glucose (2 g/kg B. wt.) after 12 h of fasting. Blood glucose was proximately determined by the glucose oxidase method using a glucose analyzer (Accu-Chek, Roche, Indianapolis, IN, USA); if blood glucose was still at a higher level than 200 mg/dL after 120 min oral glucose that defined high blood glucose. Afterwards, mice were divided into two groups (1) Group A was diet-induced glucose intolerance (DIO, *n* = 30) and Group B was fed a normal diet (Con, *n* = 5) for subsequent experiments.

#### 4.4.4. Long Term Administrations

Control and diabetes-induced mice were subjected to various treatments, Con (control, *n* = 5), DIO (diet-induced obese, *n* = 6), HCD (10 or 30 mg/kg B. wt., *n* = 6), MSN-HCD (10 mg/kg B. wt., *n* = 6) and DPP4i (10 mg/kg B. wt., *n* = 6) for 5 weeks.

#### 4.4.5. Oral Glucose Tolerance Test (OGTT)

Control and diet-induced ICR male diabetic mice were employed for this test after 12 h fasting. Mice were subjected to various treatments (Con, control, *n* = 5) and DIO (diet-induced obese, *n* = 6), HCD (10 or 30 mg/kg B. wt., *n* = 6), MSN-HCD (10 or 30 mg/kg B. wt., *n* = 6) and DPP4i (10 mg/kg B. wt., *n* = 6) by p.o. with D-glucose (2 g/kg B. wt.). At approximately 0–180 and 0–240 min, blood at every 30 min interval was sampled by venipuncture from the tail vein for determining blood glucose. Blood glucose was immediately determined by the glucose oxidase method using glucose analyzer (Accu-Chek, Roche).

#### 4.4.6. Biochemical Analysis of Blood

After five weeks of treatment, a total of 300 µL of blood was collected from piercing cheek and 100 µL of blood was employed to determine the glycated hemoglobin (HbA1c). The remaining blood was centrifuged at 888 *g* for 15 min at 4 °C. The serum was separated and analyzed for triglyceride (TG), total cholesterol (CHO), glutamate oxaloacetate transaminase (GOT) and glutamate pyruvate transaminase (GPT) using an automatic analyzer (ARTAX Menarini Diagnostics, Florence, Italy) with enzymatic colorimetric assay reagent strips (Human, Wiesbaden, Germany).

### 4.5. Statistical Analyses

All data were expressed as means with standard deviations (mean ± SD) and the data were analyzed using one-way ANOVA with the Tukey’s test. Statistical significance was defined as *p* < 0.05. All statistical procedures were performed using GraphPad Prism version 5.01 (GraphPad Software, Inc., La Jolla, CA, USA).

## 5. Conclusions

There have been numerous researches focused on drug delivery by using nanoscale carriers such as titania nanomaterials and polyalkylcyanoacrylate [26,43]. Furthermore, the nano-carriers including electrospun nanofiber [44], chitosan-coated solid lipid nanoparticle [45], and poly(lactide-*co*-glycolide) (PLGA) [46] encapsulated with anti-diabetic drug have been evaluated. Undoubtedly, the present study was the first to report the preparation of HCD-incorporated MSNs as novel anti-diabetic agents to circumvent the problems associated with natural products. The surface properties of nanoparticles were physically characterized and the results were found to be commendable. The MSN nano-vehicle has several advantages in delivering natural DPP4 inhibitor to act against diabetes with slow release of HCD and reduced levels of TG, GOT, and GTP without adverse effects. In addition, natural compound HCD and its successor MSN-HCD improved insulin resistance and promoted metabolism to control body weight as a valuable method, suggesting that they have the potential to develop as hypoglycemic drugs for diabetes therapy via DPP4 inhibition.

## Figures and Tables

**Table 1:** Structure Parameters Showing Brunauer-Emmett-Teller (BET) and dynamic light scatter (DLS) measurements of MSN Nanoparticles after Functionalization with Different Moieties.

Sample	Surface Area (m^2^/g)	Pore Volume (cm^3^/g)	Pore Size (nm)	Particle Size (nm) ^b^	Zeta Potential (mV) ^b^
MSN-NH_2_	855	1.3	3.9	168 ± 4.5	16 ± 0.4
MSN-HCD	194	0.4	N.D. ^a^	258 ± 5.7	4 ± 0.3

^a^ N.D. = Not determined; ^b^ All values are represented as mean ± SD. Mesoporous silica nanoparticles (MSN)-NH_2_; 16-hydroxycleroda-3,13-dine-16,15-olide (HCD).
